# Airway epithelial cell tolerance to *Pseudomonas aeruginosa*

**DOI:** 10.1186/1465-9921-6-26

**Published:** 2005-04-01

**Authors:** Qi Wu, Zhong Lu, Margrith W Verghese, Scott H Randell

**Affiliations:** 1 Cystic Fibrosis/Pulmonary Research and Treatment Center, Department of Medicine, The University of North Carolina, Chapel Hill, NC 27599, USA; 2 Department of Cellular and Molecular Physiology, The University of North Carolina, Chapel Hill, NC 27599, USA

## Abstract

**Background:**

The respiratory tract epithelium is a critical environmental interface that regulates inflammation. In chronic infectious airway diseases, pathogens may permanently colonize normally sterile luminal environments. Host-pathogen interactions determine the intensity of inflammation and thus, rates of tissue injury. Although many cells become refractory to stimulation by pathogen products, it is unknown whether the airway epithelium becomes either tolerant or hypersensitive in the setting of chronic infection. Our goals were to characterize the response of well-differentiated primary human tracheobronchial epithelial cells to *Pseudomonas aeruginosa*, to understand whether repeated exposure induced tolerance and, if so, to explore the mechanism(s).

**Methods:**

The apical surface of well-differentiated primary human tracheobronchial epithelial cell cultures was repetitively challenged with *Pseudomonas aeruginosa *culture filtrates or the bacterial media control. Toxicity, cytokine production, signal transduction events and specific effects of dominant negative forms of signaling molecules were examined. Additional experiments included using IL-1β and TNFα as challenge agents, and performing comparative studies with a novel airway epithelial cell line.

**Results:**

An initial challenge of the apical surface of polarized human airway epithelial cells with *Pseudomonas aeruginosa *culture filtrates induced phosphorylation of IRAK1, JNK, p38, and ERK, caused degradation of IκBα, generation of NF-κB and AP-1 transcription factor activity, and resulted in IL-8 secretion, consistent with activation of the Toll-like receptor signal transduction pathway. These responses were strongly attenuated following a second *Pseudomonas aeruginosa*, or IL-1β, but not TNFα, challenge. Tolerance was associated with decreased IRAK1 protein content and kinase activity and dominant negative IRAK1 inhibited *Pseudomonas aeruginosa *-stimulated NF-κB transcriptional activity.

**Conclusion:**

The airway epithelial cell response to *Pseudomonas aeruginosa *entails adaptation and tolerance likely mediated, in part, by down-regulation of IRAK1.

## Background

The innate immune system suppresses pathogen attachment, colonization, growth, and invasion and co-ordinates adaptive immunity [1]. Innate immunity entails recognition of microbial signatures by the cellular repertoire of Toll-like receptors (TLRs [2,3]). TLR agonists initiate receptor-specific downstream signaling pathways, ultimately enhancing production of anti-microbial molecules and inflammatory mediators [4]. Much data has been derived from monocyte/macrophages and myelocytic cell lines, but the TLR pathway also functions in epithelial cells where receptor and co-receptor expression levels, and the activity of downstream signal transduction intermediates, likely determine cellular sensitivity to pathogen products [5-7]. In the human airway, polymorphisms in TLR4, the LPS receptor, modulated the response to inhaled LPS, and TLR4 was found on primary tracheobronchial epithelial (hTBE) cells [8]. RNA for TLR1-6 was present in hTBE cells *in vitro*, and high doses of commercial LPS activated NF-κB and induced the neutrophil chemotactic cytokine IL-8 and the anti-microbial peptide beta defensin 2 (hBD-2) [9]. More recent studies demonstrate TLR2-dependent IL-8 and hBD-2 production by hTBE cells [10]. Culture filtrates of both Gram-positive and -negative bacteria and a TLR2 agonist enhanced IL-8 secretion by hTBE cells 3–5 fold [11]. *Hemophilus influenzae*, an important respiratory tract pathogen, signals via TLR2 in epithelial cells [12]. Recent studies indicate a role for TLR2 and TLR5 in stimulation of airway epithelial cells by flagellin or live Gram-positive and -negative bacteria [13,14] and TLR2 was apparently recruited to lipid rafts at the apical epithelial cell surface [15]. TLR1-10 expression and positive responses to several TLR agonists were recently reported in airway epithelial cells[16]. Thus, the TLR signal transduction pathway is likely an important regulator of airway immunity and inflammation.

The regulation of TLR signaling is dynamic. Up-regulation of TLRs, for example by interferon [17] or virus [18], may enhance epithelial cell sensitivity to pathogen products. On the other hand, LPS exposure induces hypo-responsiveness to a second challenge, termed LPS tolerance (reviewed in [19]). Other molecules acting through the TLR pathway, including mycobacterial products [20] and lipoteichoic acid from Gram-positive bacteria [21], also induce tolerance. Tolerance is associated with decreased degradation of NF-κB inhibitory proteins, reduced MAP kinase phosphorylation, prevention of NF-κB and AP-1 activation, altered transcriptional responses and suppression of pro-inflammatory cytokine and chemokine production [22]. Decreased cell surface TLR4 protein [23] was associated with tolerance, but tolerance could not be attributed solely to receptor loss, since cells that did not decrease TLR4 still became tolerant, and over-expression of CD14, TLR4 and MD-2 in HEK293 cells did not prevent LPS tolerance [20,24]. However, a hypo-responsive state can be induced at the level of the plasma membrane by the expression of endogenous, functionally inactive members of the Toll-interleukin 1 receptor superfamily [25]. Many substances acting though the TLR signal transduction pathway induce cross tolerance, including LPS and IL-1β [22], LPS and mycobacterial products [20], or LPS and lipoteichoic acid [21]. Tolerance without down-regulation of surface receptors and cross-tolerance suggest negative regulation of common elements in the downstream signal transduction pathway. IRAK1 functions just distal to TLRs and their adaptor proteins [3], and tolerance is associated with decreased IRAK1 protein many cell types [26-31]. Alternatively, signaling through IRAK1 may be impaired due to decreased TLR4-MyD88 complex formation [32], lack of dissociation from the receptor complex [33], or increased function of inhibitory forms of IRAK such as IRAK-M [34]. Hypo-responsiveness may also be due to events closer to activation of transcription factors, for example, abrogation of IκBα polyubiqitination [35], over-expression of unique IκB inhibitory proteins [36], or production of NF-κB p50 homo-dimers [37], or other factors that block NF-κB DNA binding [38]. These diverse negative regulatory processes may be generally important to protect the host from overly exuberant, destructive inflammatory responses.

In cystic fibrosis (CF), the lack of functioning CFTR impairs mucociliary and cough clearance [39], forming a nidus for infection and allowing organisms such as *Pseudomonas aeruginosa *(Ps. a.) to evade host defenses. Inability to clear infected mucus results in continuous exposure of airway epithelial cells to bacteria and their products. Ongoing host-pathogen interactions determine the extent of the inflammatory response, and in turn, rates of tissue destruction and loss of pulmonary function. Strategically located between luminal bacterial masses and the host circulation, the airway epithelium is in a key position to regulate inflammation, but it remains unknown whether the airway epithelium becomes either hypersensitive or tolerant to the chronic presence of bacterial products. Our goals were to characterize the response of well-differentiated, primary hTBE cells to an apical surface challenge with Ps. a. products, to determine whether repeated exposure induced tolerance and, if so, to explore the mechanism(s). We show that Ps. a. products activate the TLR pathway and that hTBE cells become tolerant via a mechanism likely involving down-regulation of IRAK1.

## Methods

### Reagents

Antibodies against phosphorylated or total c-jun NH_2_-terminal kinases (JNK), p38, extracellular signal-regulated kinases (ERK), and total IκBα were from Cell Signaling Technology (Beverly, MA). Antibody against IRAK1 was from Upstate Biotechnology, Inc. (Lake Placid, NY). Anti-NF-κB p50 and p65 subunits were from Santa Cruz Biotechnology (Santa Cruz, CA). An ELISA kit for IL-8 (DuoSet ELISA Development System) was from R&D Systems (Minneapolis, MN). An *in vitro *toxicology assay kit for lactate dehydrogenase (LDH) was from Sigma (St. Louis, MO), as were other standard reagents unless otherwise specified.

### Preparation of Ps. a. filtrate

Ps. a. strain ATCC 27853 was grown in trypticase soy broth (TSB) for 72 hours at 37°C with shaking at 250 RPM. Following centrifugation at 5,500 × G (4°C) for 30 minutes, the supernatant was 0.45 μm filtered, aliquoted and stored at -20°C. TSB treated similarly was used as a control. When used in experiments with cell lines cultured on plastic, Ps. a. filtrates or TSB were boiled for 10 minutes to eliminate protease activity. Consistent with prior reports [40], we found IL-8 stimulatory activity in Ps. a. filtrates to be heat-resistant.

### Cell culture

Under an Institutional Review Board-approved protocol, hTBE cell cultures were prepared as previously described[41]. Briefly, epithelial cells were removed from the lower trachea and bronchi by protease XIV digestion and cells were plated in BEGM medium on collagen-coated dishes. Passage 2 cells were cultured on type VI collagen (Sigma) coated Millicell CM inserts (0.4 μM pore size, Millipore Corporation, Bedford, MA) in ALI medium. The cell seeding density for 10 mm and 30 mm diameter inserts was 0.15 × 10^6 ^and 1 × 10^6 ^cells per insert, respectively. Following confluence after 5–7 days, cultures were maintained with an air-liquid interface until well-differentiated and were used at 21 days. Endotoxin in ALI medium was less than 100 pg/ml (LAL assay, Bio-Whittaker, Walkersville, MD). A recently described immortalized cell line, referred to as AALEB, was derived from hTBE cells by infection with retroviruses expressing SV40 early region and telomerase reverse transcriptase [42]. AALEB cells were grown on plastic dishes in BEGM medium under standard culture conditions.

### Ps. a. filtrate challenge

Experiments with well-differentiated hTBE cells on 12 and 30 mm Millicell inserts were performed in 12 or 6 well plates, respectively. Before challenge, the apical culture surface was rinsed once with Dulbecco's PBS. Ps. a. filtrate in ALI medium supplemented with 10% human serum (Sigma #H4522) was added to the apical culture surface, using 100 μl for 12 mm inserts and 500 μl for 30 mm inserts. One or two ml of ALI medium was added to the basolateral side of the 12 or 6 well plates, respectively, and the cultures were incubated at 37°C in 5% CO_2 _for 24 hours. Following removal of basolateral medium for IL-8 or LDH assay, the apical and basolateral surfaces were washed twice with PBS. After incubation at 37°C for 1–2 hours with fresh basolateral ALI medium, the cultures were re-challenged apically with Ps. a. filtrate plus serum as described above. Challenges with IL-1β (10 ng/ml) or TNFα (25 ng/ml) were performed in ALI medium without serum. IL-8 and LDH assays were performed with commercial kits as specified previously [11] and are based on results from triplicate wells using cells from at least three different individuals, unless stated otherwise.

### Western blot analysis

At specified times following challenge, cells were harvested from 30 mm inserts into ice-cold lysis buffer (100 mM TrisHCl pH 8.0, 100 mM NaCl, 5.0 mM NaF, 2 mM EDTA, 1% NP-40, 1 mM Na_3_VO_4_, 100 μM TPCK, 100 μM quercetin, 1 mM PMSF, 1 μg/ml leupeptin, and 1 μg/ml pepstatin) using a cell scraper, transferred to tubes and set on ice for 20 minutes. Following centrifugation, protein concentrations were determined using the BCA Protein Assay Reagent (Pierce, Rockford, IL). Samples were resolved by SDS-PAGE (4–20% tris-glycine gels, Invitrogen, San Diego, CA) and blotted onto Immobilon-P membranes (Millipore Corp., Bedford, MA). Blots were blocked in TBS with 0.05% Tween 20 and 5% dry milk powder, incubated with primary then secondary antibodies (Jackson ImmunoResearch Laboratories, Inc., West Grove, PA) followed by chemiluminescence detection of peroxidase (Pierce).

### Nuclear extracts

Cells were scraped from 30 mm inserts into 0.8 ml ice-cold PBS containing protease inhibitors (1 μM PMSF, 1 μg/ml leupeptin, 1 μg/ml pepstatin, and 0.5 μM DTT) and were centrifuged. The cell pellet was re-suspended in buffer (10 mM Hepes, pH 7.9, 10 mM KCl, 1.5 mM MgCl_2_, 0.2 mM EDTA, 0.5 mM DTT, 0.25% NP-40, 1 μM PMSF, 1 μg/ml leupeptin and 1 μg/ml pepstatin) on ice for 10 minutes and cells were lysed with a Dounce homogenizer (Kontes Scientific Glassware, Vineland, NJ). The nuclei were extracted with high salt buffer (20 mM Hepes, pH 7.9, 450 mM NaCl, 1.5 mM MgCl_2_, 0.2 mM EDTA, 0.5 mM DTT, 25% glycerol, 1 μM PMSF, 1 μg/ml leupeptin and 1 μg/ml pepstatin) on ice for 20 minutes with occasional vortexing. Supernatants were prepared by centrifugation and protein concentration was determined as above.

### Electrophoretic mobility shift assays

NF-κB-specific consensus oligonucleotide (5' AGTTGAGGGGACTTTCCCAGGC3') and AP1-specific consensus oligonucleotide (5' CGCTTGATGAGTCAGCCGGAA3') were from Promega (Madison, WI). DNA probes were ^32^P end labeled with T4 polynucleotide kinase (Promega). Nuclear extracts (2.5 μg) were incubated with 40,000–60,000 cpm of ^32^P end labeled oligonucleotide probe in binding buffer (final volume of 10 μl) containing 1 μg poly dI-dC (Sigma), 10 mM TrisHCl, pH 7.9, 50 mM KCl, 1 mM DTT, 0.25 mg/ml BSA, 4% glycerol for 20 minutes at room temperature. For supershift analysis, nuclear extracts were preincubated with 0.5 μl of antisera against NF-κB p50 or p65 sub-units for 10 minutes in binding buffer. Unlabeled NF-κB, AP-1 or SP1 (5' ATTCGATCGGGGCGGGGCGAGC3') oligonucleotides were used as competitors. Complexes were separated on 5% non-denaturing polyacrylamide-urea gels, which were dried and exposed to a PhosphorImager screen (Amersham Pharmacia Biotech, Piscataway, NJ).

### IRAK1 in vitro kinase assay

Cells were harvested from 30 mm inserts into 600 μl ice-cold lysis buffer (50 mM HEPES, pH 7.6, 150 mM NaCl, 1 mM EDTA, 1% NP-40, 1 mM Na_3_VO_4_, 20 mMβ-glycerophosphate, 1 mM NaF, 1 mM benzamidine, 5 mM para-nitrophenylphosphate, 1 mM DTT, 1 mM PMSF, 1 μg/ml leupeptin, 1 μg/ml aprotinin, and 1 μg/ml pepstatin) using a cell scraper. After brief vortexing and incubation on ice for 20 minutes, tubes were centrifuged and supernatant protein concentrations were determined as above. For immunoprecipitation, 1000 μg of protein extract was precleared with 2 μg of normal rabbit IgG and 20 μl of protein G-agarose slurry. Protein G beads were pelleted and the supernatant was incubated with 2 μg of rabbit IgG against IRAK1. Protein G-agarose slurry (20 μl) was added and incubated for 1 hour and the beads were washed 3 times with lysis buffer and twice with kinase buffer without ^32^P ATP (see below). Beads were suspended in 20 μl of kinase buffer (20 mM Tris-HCl, pH 7.6. 20 mM MgCl_2_, 20 mM β-glycerophosphate, 1 mM benzamidine, 20 mM para-nitrophenylphosphate, 0.4 mM PMSF; 1 mM sodium metabisulfite, 2 μM cold ATP and 10 μCi γ-^32^P ATP). Reactions were allowed to proceed at 30°C for 30 min and terminated with SDS sample buffer. Samples were then run on 4–20% polyacrylamide gels, dried, and exposed to a PhosphorImager screen as above.

### NF-κB reporter assay and expression of dominant negative IRAK1

Adenoviral vectors constitutvely expressing the LacZ gene from the CMV promoter (Ad.CMV-lacZ) and NF-κB-responsive firefly luciferase (Ad.NF-κB-fLuc) have been described previously [43]. We created an adenoviral vector expressing the DD domain of IRAK1 (NCBI accession # NM_001569, amino acids 1–80), which is reported to function as a dominant negative (dn) [44]. A plasmid encoding human IRAK1 was kindly provided by Dr. X. Li (The Cleveland Clinic, Cleveland, OH) and was used as a template for PCR using primers (forward: 5'-CTC GAG GTG CCA GGC TGT GA-3', reverse: 5'-GCT AGC CGG CAG CCA TGG-3') adding 5' and 3' XhoI and NheI sites, respectively. The amplified fragment was cloned into the pCR2.1 vector (Invitrogen). The resulting plasmid was digested with XhoI /NheI and the fragment was ligated into XhoI /NheI-digested pShuttle-IRES-hrGFP-1 adenoviral expression vector plasmid (Stratagene, LaJolla, CA). This strategy placed the DD domain of IRAK1 in frame with the 3X Flag tag of the vector, and the final construct was verified by sequencing. Adenoviral vectors were created, plaque-purified and amplified using conventional methods. AALEB or hTBE cells were transfected with Ad.CMV-lacZ and Ad.NF-κB-fLuc and pShuttle-IRES-hrGFP-1 empty vector or pShuttle-dnIRAK1-IRES-hrGFP-1, using a 1:10 ratio of reporter and expression vectors, respectively. Cells were exposed to viruses for 2 hours, 48 hours prior to experimental challenge, using transient permeabilization [45] for hTBE cells. Eight hours after challenge, cells were lysed and fLuc and β-galactosidase activity measured as described previously [46,47].

## Results

### The initial response of hTBE cells to Ps. a. products

**Figure 1:**
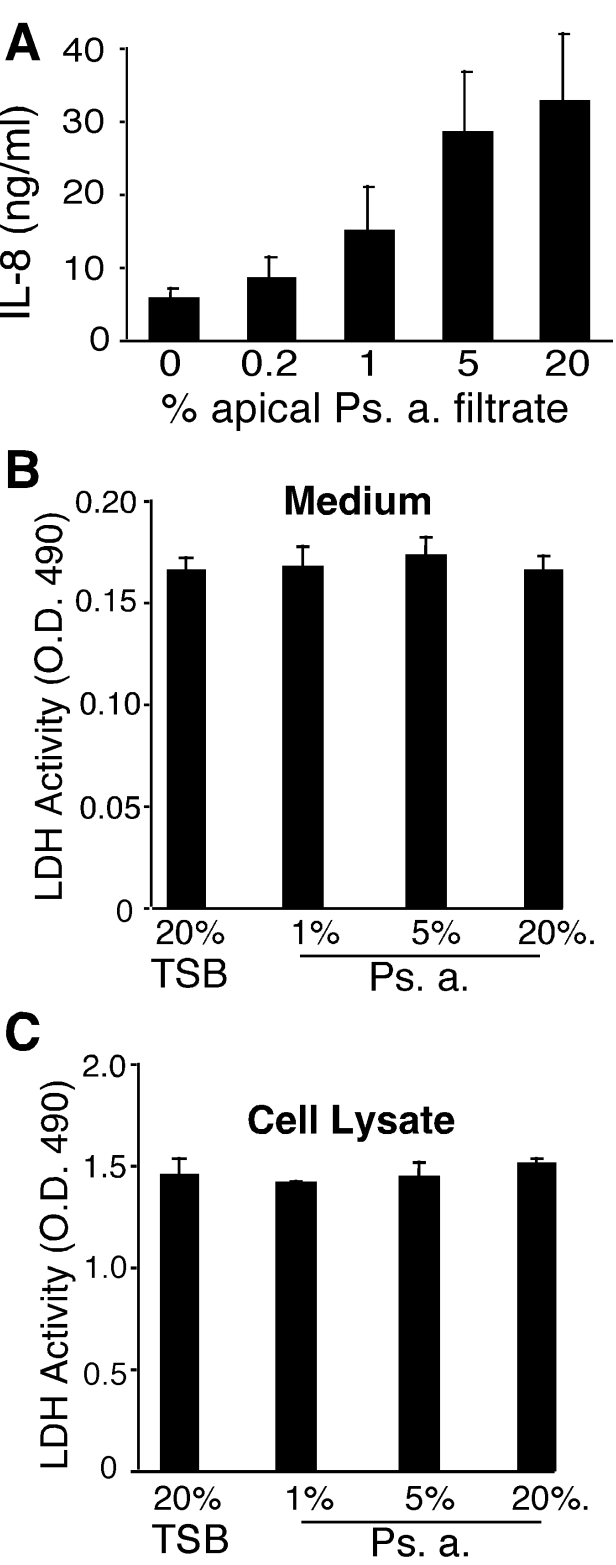
**Treatment of well-differentiated hTBE cells with Ps. a. culture filtrates induced IL-8 secretion and was non-toxic. **A) The apical surface of well-differentiated hTBE cell culures was challenged with the indicated concentration of Ps. a. filtrate or 20% TSB as a control (0% Ps. a. group) in the presence of 10% human serum, and IL-8 was measured in the 24-hour conditioned basolateral medium. The results are the mean + SEM from 3 independent experiments with cell cultures from 3 different donors. B and C) Ps. a. filtrate was essentially non-toxic as illustrated by lack of LDH secretion into the medium and retention of cellular LDH. The results are from three replicate wells (mean + SD).

To simulate *in vivo *host-bacterial product interactions, we challenged the apical surface of polarized, well-differentiated hTBE cells with late stationary phase Ps. a. filtrates. Exposure of the apical culture surface to 20% TSB (the negative control bacterial broth) in the presence of 10% human serum added as a source of LPS binding protein and soluble CD14, for 24 hours, caused a modest, < 1 fold, culture-dependent increase in IL-8 secretion ([11] and data not shown). Challenge with Ps. a. filtrate induced IL-8 secretion as a function of dose (Figure 1A), causing an average 6.3 ± 1.4 fold increase over the TSB control at the 20% Ps. a. filtrate dose (mean ± SEM, cells from 3 independent donors). We have previously shown that Ps. a. treatment similarly induces IL-6, but not IL-10 or RANTES [11]. Ps. a. challenge of well-differentiated hTBE cultures was non-toxic, as assessed by lack of LDH release into the medium (Figure 1B and 1C) and maintenance of a patent air-liquid interface [for more information, including a positive control, see the online supplement of reference [11], ].

**Figure 2:**
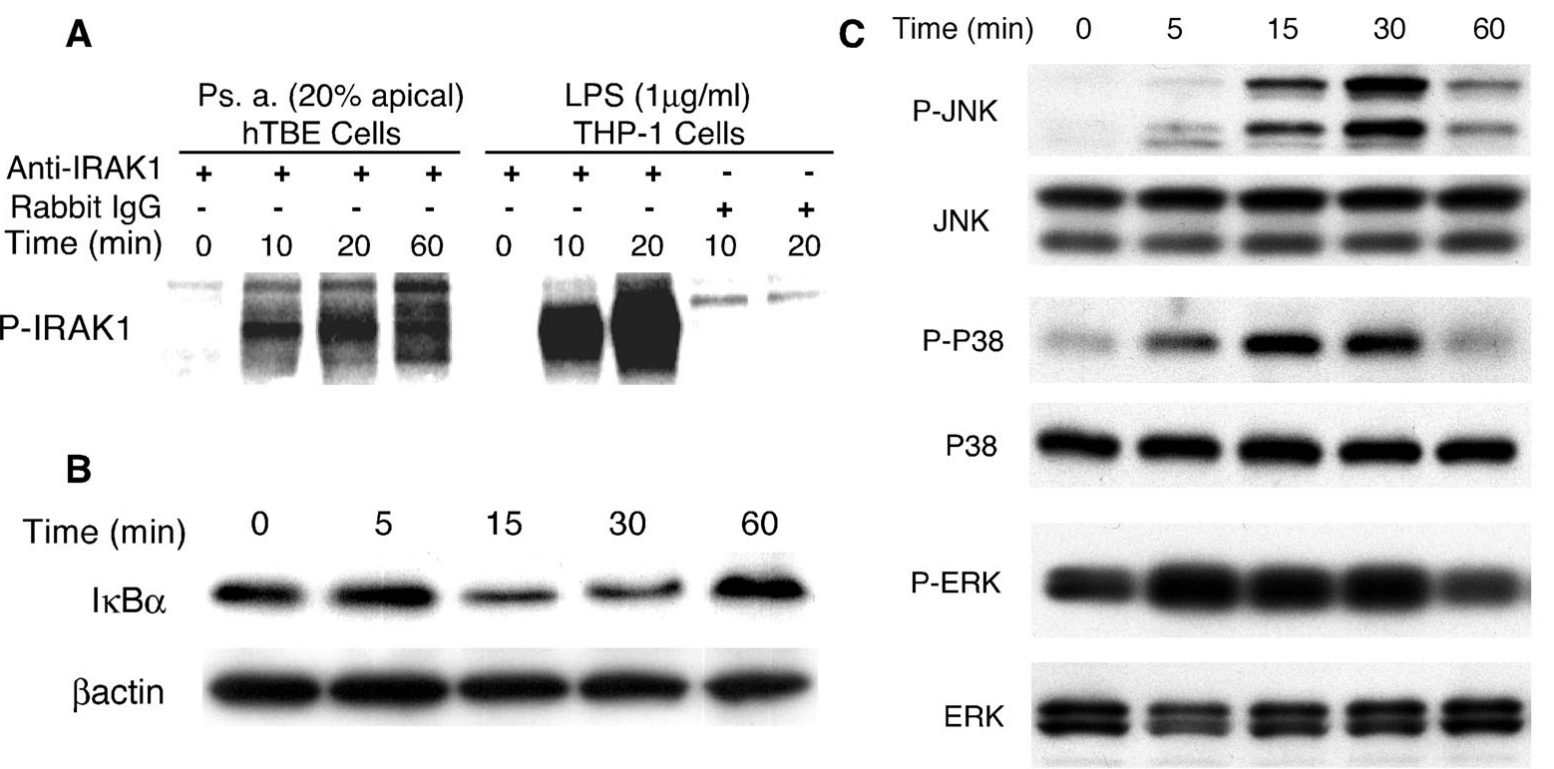
**Ps. a. filtrate induced IRAK1 autophosphorylation, IκBα degradation and MAP kinase phosphorylation. **A)Proteins were harvested from day-21 hTBE cell cultures treated apically with 20% Ps. a. filtrate or from THP1 monocytic cells treated with LPS at the times indicated. Equal amounts of protein were immunoprecipitated with anti-IRAK1 or control IgG for each cell type. Precipitates were subjected to *in vitro *kinase assay and polyacrylamide gel electrophoresis and exposed to a Phosphoimager screen. B and C) Day-21 hTBE cell cultures were treated with Ps. a. filtrate, and harvested for western blot of IκBα (B) or phospho- and total MAP kinases (C) at the indicated time points. Equal amounts of protein were run per lane on each gel. The βactin and total MAP kinase western blots represent re-probing of the IκBα and phospho-MAP kinase blots, respectively. The results are representative of 3 separate experiments with cells derived from 3 different donors.

**Figure 3:**
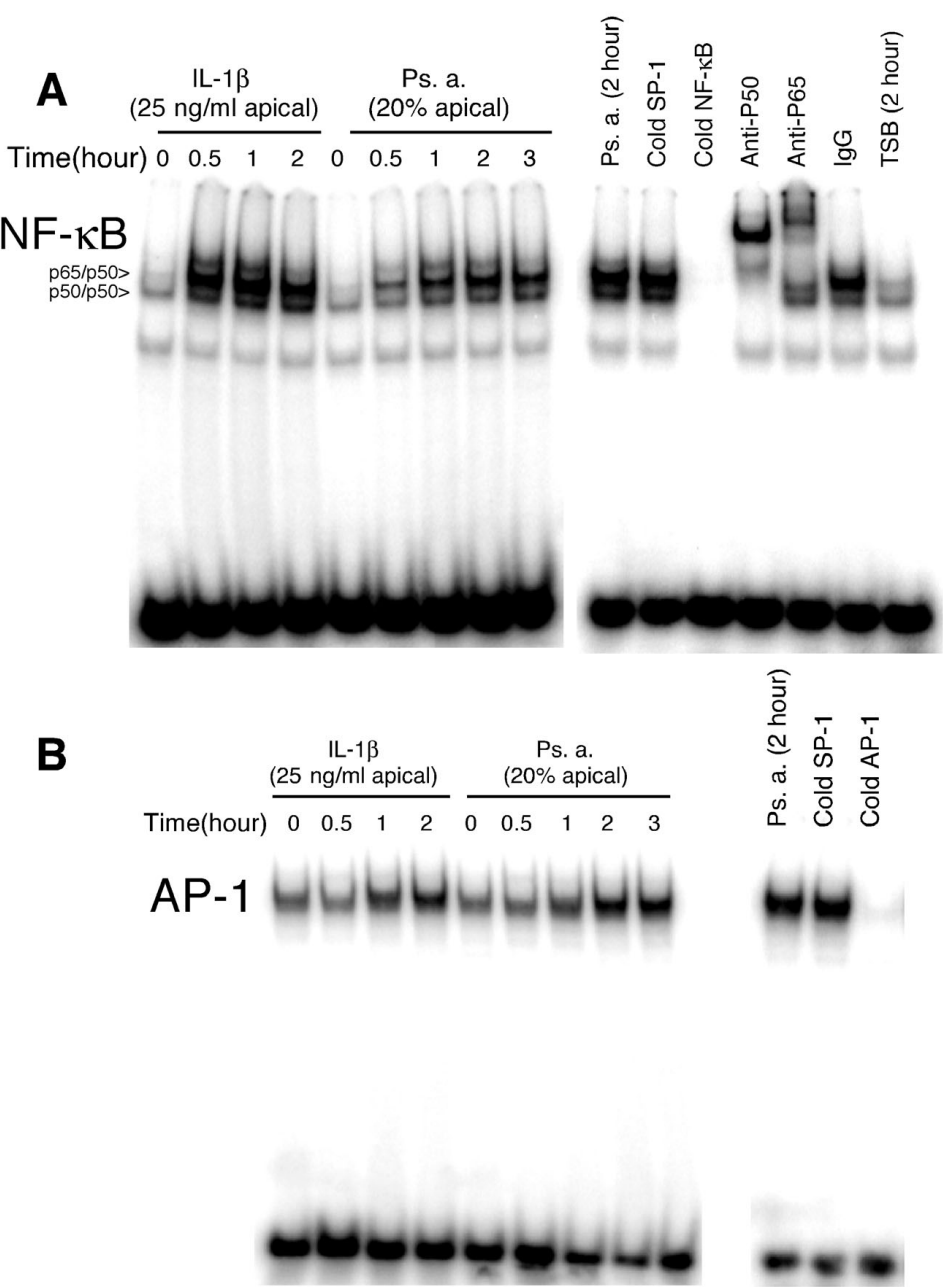
**Ps. a. filtrate increased nuclear content of NF-κB and AP-1 transcription factors. **Nuclear extracts were prepared from day-21 hTBE cell cultures challenged apically with Ps. a. filtrate or IL-1β at the times noted and equal amounts of nuclear protein per lane were subjected to EMSA using oligonucleotide probes for NF-κB (A) or AP-1 (B). The inhibitor and supershift assays were all performed with nuclei extracted 2 hours after TSB or Ps. a. challenge. The results are representative of 3 separate experiments with cells derived from 3 different donors.

In several cell types, inflammatory mediator synthesis following bacterial product challenge is regulated by TLR activation of NF-κB and AP-1 transcription factors. We determined whether Ps. a. filtrates stimulated the TLR signal transduction pathway in well-differentiated hTBE cells. Challenge of hTBE cultures with 20% Ps. a. filtrate promptly induced IRAK1 phosphorylation (Figure 2A), IκBα protein degradation (Figure 2B), and MAP kinase phosphorylation, including JNK, p38, and ERK (Figure 2C). On the basis of equivalent protein input to the immunoprecipitaiton reaction, the LPS-treated THP-1 monocytic cells, used as positive and negative controls, contained much more phospho-IRAK1 than hTBE cells. We did not reduce the concentrations of growth factors in the culture media prior to challenge thus, ERK phosphorylation was relatively high at baseline. Nonetheless, we still observed enhanced ERK phosphorylation after Ps. a. challenge. As predicted, IκBα degradation and MAP kinase activation preceded elevated nuclear content of NF-κB and AP-1 transcription factors, respectively (Figure 3A and 3B). The predominant shifted NF-κB band was composed of p50/p65 heterodimers. We did not attempt supershift assays to identify the subset of AP-1 transcription factors. IRAK1 phosphorylation and stimulation of the NF-κB and MAP kinase pathways is consistent with Ps. a. filtrate activation of the MyD88-dependent component of the TLR receptor signal transduction pathway in hTBE cells.

### The response of hTBE cells to repeated Ps. a. challenge

**Figure 4:**
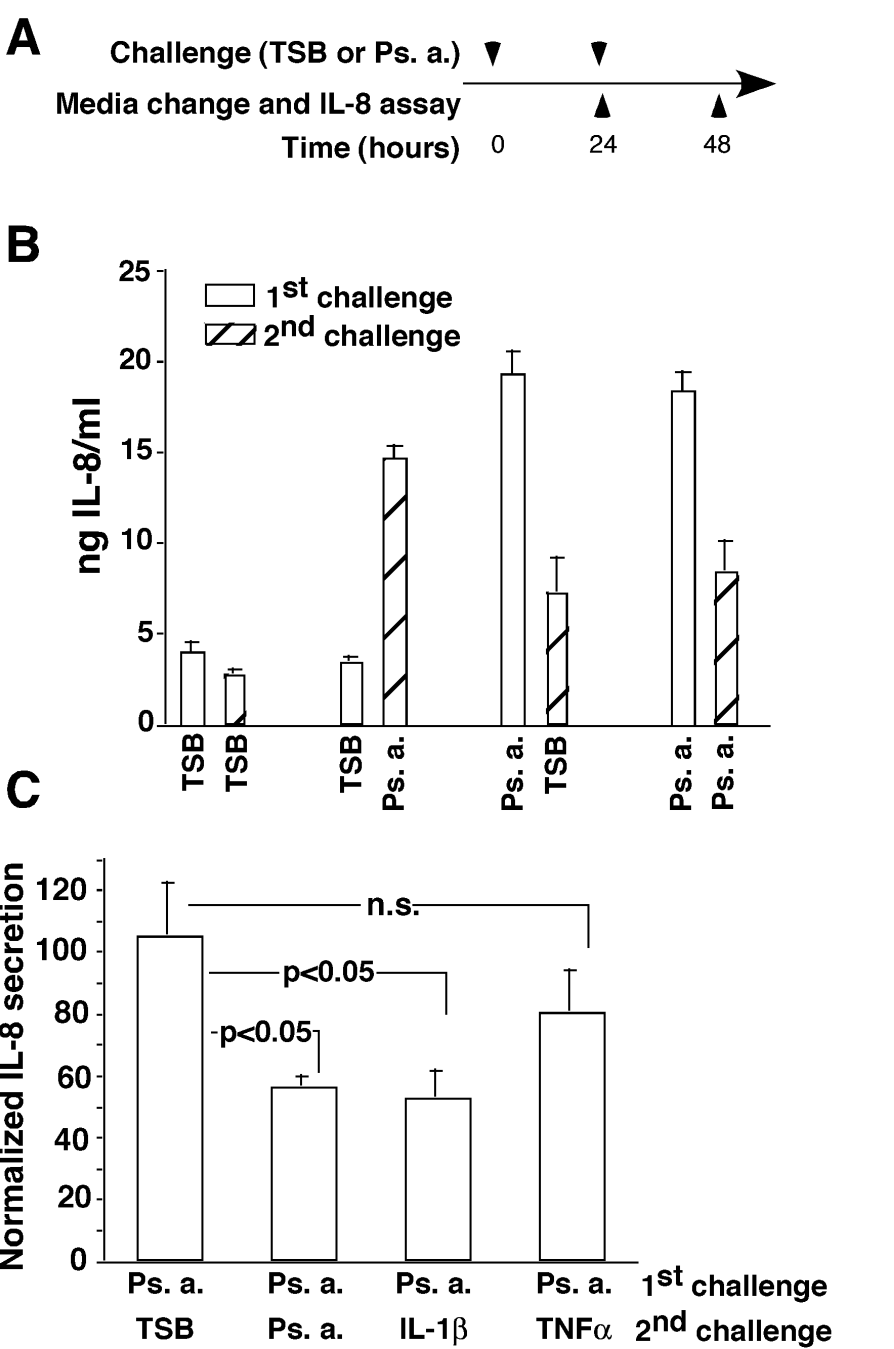
**Ps. a. filtrate induced tolerance to a second Ps. a. challenge and cross-tolerance with IL-1β but not TNFα. **The apical surface of well-differentiated, day-21 hTBE cell cultures was challenged with Ps. a. filtrate or TSB as schematically illustrated in (A) and IL-8 production was measured in the basolateral medium at 24 or 48 hours. The results of a representative experiment are given as means ± SD in panel (B) and are from duplicate assays of three replicate wells. Similar results were obtained in 3 separate experiments with cell cultures from 3 different donors. C) The apical surface of well-differentiated, day-21 hTBE cell cultures (triplicate wells) was challenged with Ps. a. filtrate or TSB. Following washing and basolateral media change, the cells were re-challenged as indicated. IL-8 production during the second 24 hour period was normalized by dividing the Ps. a.-pretreated IL-8 value by the corresponding value in TSB-pretreated cultures (eg. Ps. a. → Ps. a./TSB → Ps. a.). The results are the average ± SEM of three independent experiments using cells from different donors. P values are based on ANOVA and Tukeys test, n.s. = not significant.

To assess whether prior exposure to Ps. a. filtrates induced tolerance, we re-challenged hTBE cell cultures 24 hours after the initial exposure, studying the 4 possible combinations (Figure 4A). As illustrated in figure 4B, initial treatment with TSB caused minimal IL-8 secretion and no change following a second TSB treatment. Initial TSB treatment followed by Ps. a. resulted in enhanced IL-8 production during the second 24-hour period. Initial exposure to Ps. a. induced a five-fold increase in IL-8 secretion and when these cells were re-challenged with TSB they displayed lesser IL-8 secretion, but still elevated over the initial TSB control. Importantly, cells initially challenged with Ps. a. and re-challenged with Ps. a. showed less stimulation of IL-8 secretion during the second 24-hour period, almost identical to that of TSB-re-challenged cells and, thus, were tolerant. Tolerance was consistently observed, and IL-8 production by cells from 3 different individuals during the second 24 hour period was normalized by dividing the *Ps. a*. -pretreated IL-8 value by the corresponding value in TSB-pretreated cultures (Fig 4C). This analysis confirmed no significant differences in IL-8 secretion during the second 24-hour period between Ps. a. and TSB treated cells that were pre-exposed to Ps. a. filtrate. Re-challenge of Ps. a.-exposed cells with Ps. a. reduced IL-8 secretion by an average of 44 ± 3% (p < 0.05) compared to TSB pretreated cells. Interestingly, Ps. a. induced significant tolerance to a subsequent stimulation with IL-1β (47 ± 9 % reduction; p < .05), but not TNFα (20 ± 14 % reduction; p > .05). Hetero-tolerance between Ps. a. and IL-1β, but not TNFα, suggests that tolerance occurs at a point in common to the TLR and IL-1β pathways, but upstream of the convergence with the TNFα pathway (see Discussion).

**Figure 5:**
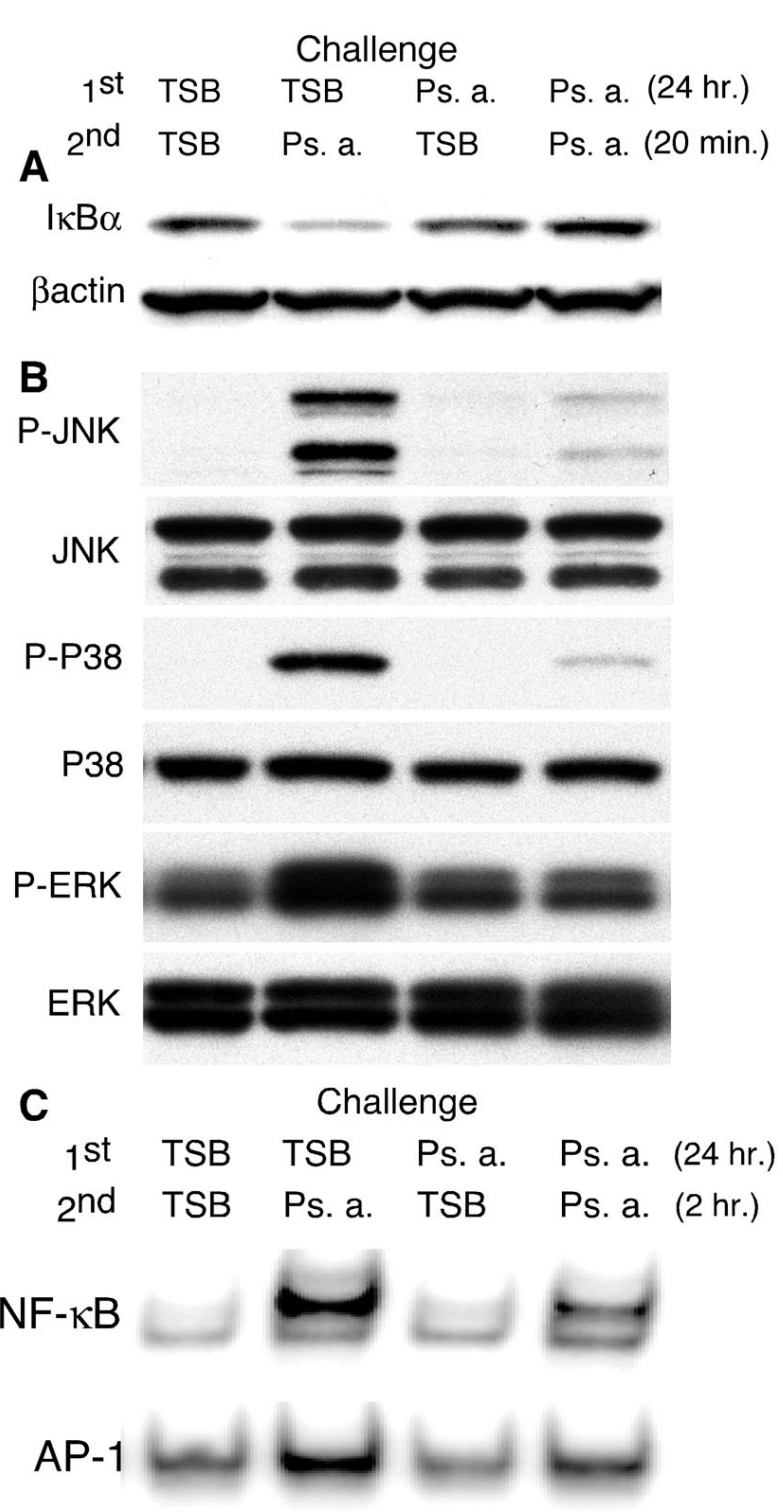
**IκBα degradation, MAP kinase activation and induction of nuclear NF-κB or AP-1 transcription factors were strongly attenuated following a second Ps. a. filtrate challenge of tolerant hTBE cells. **The apical surface of well-differentiated, day-21 hTBE cell cultures was challenged with Ps. a. filtrate or TSB as indicated and cultures were incubated for 24 hours. Following washing and basolateral media change, the apical surfaces were re-challenged as indicated and proteins were harvested for IκBα and βactin (A) or phospho- and total MAP kinase (B) western blot analysis 20 minutes later. Equal amounts of protein were run per lane on each gel. The βactin and total MAP kinase western blots represent re-probing of the IκBα and phospho-MAP kinase blots, respectively. C) hTBE cells were re-challenged as indicated and nuclear proteins were harvested 2 hours following the second challenge. Equal amounts of nuclear protein per lane were subjected to EMSA using oligonucleotide probes for NF-κB or AP-1, only the shifted bands are shown. Similar results were obtained in 3 separate experiments with cell cultures from 3 different donors.

To explore the mechanism of hTBE cell tolerance to Ps. a. products, we studied IκBα degradation, MAP kinase phosphorylation, and generation of nuclear NF-κB and AP-1 binding activity at an optimal time following the second challenge based on the prior time course studies. As shown in figure 5A and 5B, pre-exposure to Ps. a. for 24 hours largely prevented IκBα degradation and phosphorylation of JNK, p38 and ERK MAP kinases in response to a second Ps. a. challenge, while cells pre-exposed to TSB responded vigorously. Likewise, pre-exposure to Ps. a. prevented the activation of NF-κB or AP-1 transcription factors (Figure 5C).

### IRAK-1 as a critical determinant of hTBE cell sensitivity to Ps. a. 

**Figure 6:**
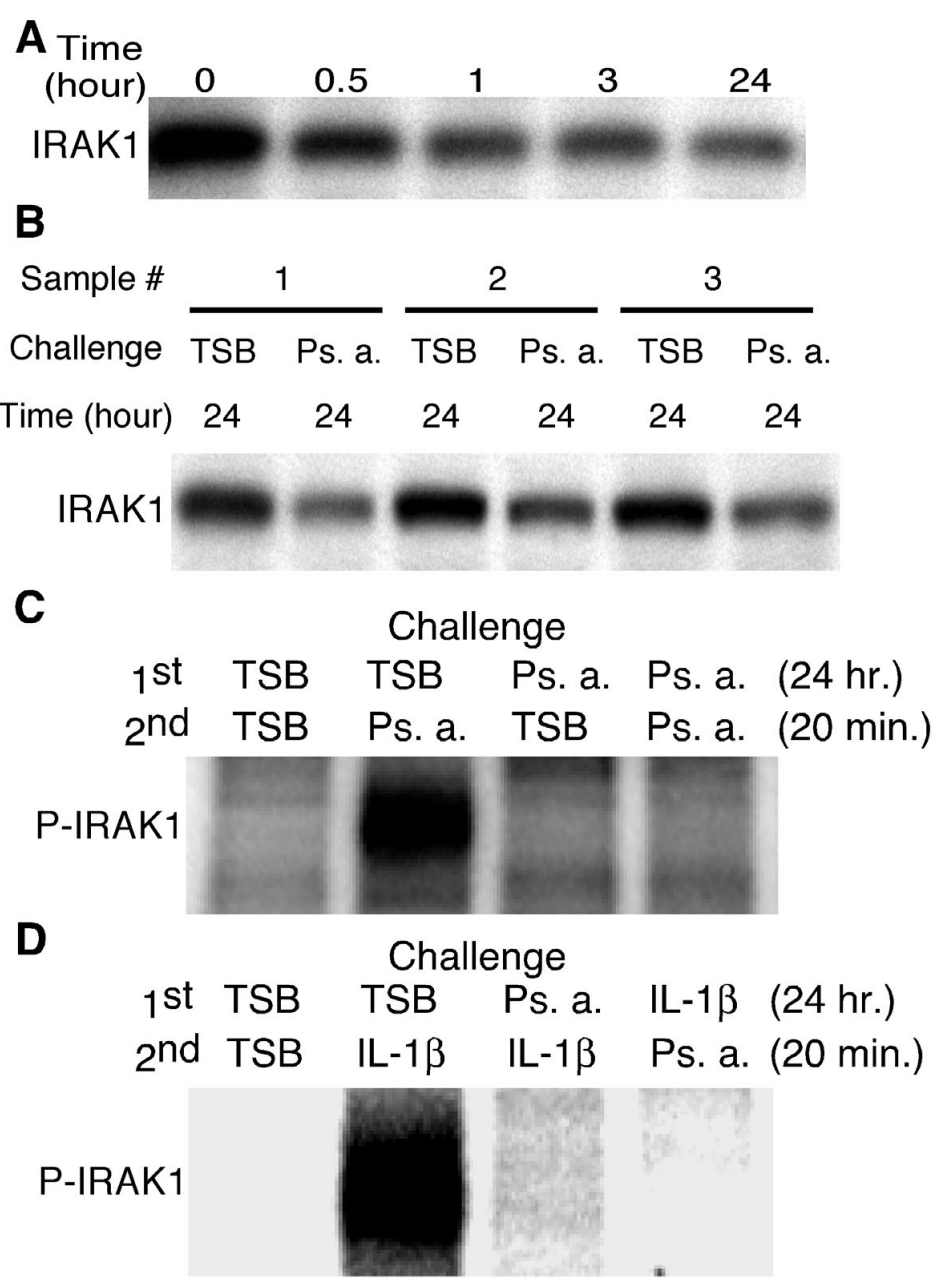
**IRAK1 protein content was reduced in tolerant hTBE cells and remaining IRAK1 was not autophosphorylated after Ps. a. or IL-1β treatment. **A) Proteins were harvested from day-21 hTBE cell cultures following apical challenge with Ps. a. filtrate at the times indicated. Equal amounts of protein were run per lane and subjected to western blot for IRAK1. B) Protein samples were obtained from cultures representing 3 different donors and IRAK protein content was examined as described in A, above. C and D) The apical surface of well-differentiated, day-21 hTBE cell cultures was challenged with Ps. a. filtrate, TSB or IL-1β as indicated and cultures were incubated for 24 hours. Following washing and basolateral media change, the apical surfaces were re-challenged as indicated and cellular protein was harvested 20 minutes following the second challenge. Equal amounts of protein per lane were immunoprecipitated with anti-IRAK1. Precipitates were subjected to *in vitro *kinase assay, run on polyacrylamide gels and exposed to a Phosphoimager screen. The results in panels C and D are representative of 3 separate experiments using cells derived from 3 different donors.

IL-1β, but not TNFα hetero-tolerance and the elimination of both MAP kinase and NF-κB responses in tolerant hTBE cells focused our attention on upstream elements of the signal transduction pathway. Prior studies have suggested that decreased IRAK1 protein is associated with LPS tolerance [26-31]. As illustrated in Figure 6A and 6B, IRAK1 protein content decreased during the initial stimulation and was reduced 62% ± 4% (mean ± SEM, n = cell cultures from 3 different individuals) at 24 hours following the initial Ps. a. challenge. Studying the same four exposure permutations described above, we found that IRAK1 remaining 24 hours following the initial Ps. a. challenge could not be autophosphorylated after a second Ps. a. challenge (Figure 6C). Thus, tolerance to Ps. a. products in hTBE cells was associated with reduced IRAK1 protein content and kinase activity. Furthermore, similar to the previously noted hetero-tolerance in IL-8 secretion between Ps. a. filtrate and IL-1β (Figure 3C), prior exposure to Ps. a. prevented IRAK1 phosphorylation in response to IL-1β, and vice versa (Figure 6D).

**Figure 7:**
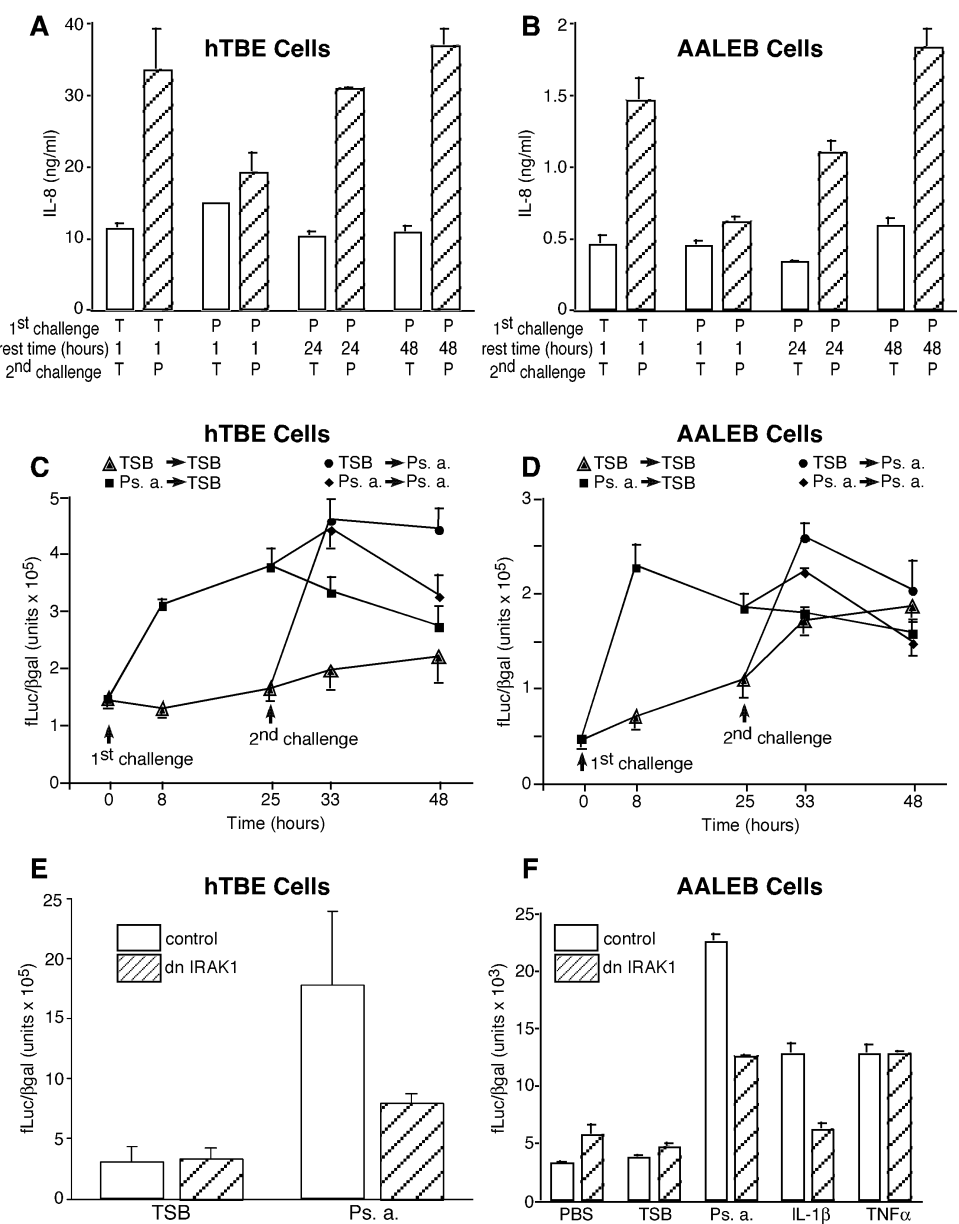
**Tolerance and loss of tolerance in IL-8 secretion and NF-κB driven transcription is similar in hTBE cells and the AALEB cell line, and dnIRAK1 inhibits responses to Ps. a. and IL-1β but not TNFα. **A-D) The apical surface of well-differentiated, day-21 hTBE cell cultures (A, C) or AALEB cells in conventional culture on plastic (B, D) were challenged with boiled Ps. a. filtrate (P) or TSB (T) in the absence of human serum as indicated. In A and B, cultures were incubated for 24 hours, and following washing and media change, the apical surfaces were re-challenged when indicated. IL-8 was measured in the media 24 hours following the second challenge. C and D) Cells were transfected with adenoviral vectors expressing constitutive or NF-κB driven reporter genes as described in Methods and were challenged with TSB or Ps. a. and lysed for reporter gene assay as indicated. Note the steep slope of the Ps. a. response in naive or TSB-pretreated cells versus the attenuated slope in Ps.a.-pretreated cells. E, F) hTBE and AALEB cells were transfected with adenoviral vectors expressing constitutive and NF-κB driven reporter genes and dnIRAK1 or a control vector as described in the Methods and were lysed for reporter gene assay 8 hours after treatment as indicated. dnIRAK1 inhibited approximately 50% of the Ps. a. response in both cell types, and, in AALEB cells, >90% of the IL-1β response, but not the TNFα response. All points represent the mean of triplicate wells ± SD. The results in panel E ands F are representative of 2 and 3 separate experiments, respectively

To facilitate molecular approaches towards understanding tolerance mechanisms in airway epithelial cells, we studied AALEB cells, a novel SV40 and telomerase immortalized hTBE derived cell line [42]. AALEB cells on plastic detached from the culture dishes when exposed to unboiled Ps. a. filtrates, presumably due to protease activity. Since prior studies demonstrated that Ps. a.-derived IL-8 stimulatory activity for A549 cells was heat resistant [40], we tested boiled Ps. a. filtrates on both hTBE and AALEB cells. Similar to unboiled filtrates in the presence of serum, hTBE cells challenged with 20% boiled Ps. a. filtrate, without serum, secreted IL-8 and developed tolerance (Figure 7A). Removal of the Ps. a. stimulus, followed by PBS washing and media change, and a 24 or 48 hour "rest time" promoted re-sensitization of hTBE cells. Although the ratio of cells to media, and thus absolute IL-8 levels, are much lower in conventional plastic cultures of AALEB cells than in air-liquid interface cultures of hTBE cells, tolerance and loss of tolerance regarding IL-8 secretion was almost identical (Figure 7B). Thus, the AALEB cell line is apparently a good model to study tolerance in airway epithelial cells.

To examine the effects of Ps. a. on NF-κB dependent gene transcription, we transfected hTBE and AALEB cells with adenoviruses expressing NF-κB-driven luciferase and constitutively expressed LacZ reporter genes. Transient permeabilization with caproic acid [45] was necessary for efficient gene transfer to hTBE cells. Luciferase activity normalized for β gal was measured at baseline and 8 and 24 hours following stimulation with the 4 possible combinations of Ps. a. or TSB. Ps. a. strongly simulated NF-κB-driven luciferase activity in naive or TSB pre-treated hTBE or AALEB cells, but the response was strongly attenuated in Ps. a. pretreated cells (compare the slopes of the 0–8 hour and 25–33 hour groups in Figure 7C and 7D). These results suggest a strong NF-κB driven transcriptional component of Ps. a. stimulation that was inhibited in tolerant airway epithelial cells.

Having established a reporter assay in a relevant cell type, it was now possible to examine whether IRAK1 down-regulation was correlative or causal in airway epithelial tolerance. AALEB cells were transfected with a 1:10 ratio of adenoviral particles expressing both reporter genes and dnIRAK1 [44] or the empty vector control, respectively. In both hTBE and AALEB cells, NF-κB driven luciferase activity was approximately 5-fold greater in response to Ps. a. in cells infected with the control, empty vector. Luciferase activity was reduced significantly (52% in hTBE cells, n = 2 independent experiments; 55 ± 8 % in AALEB cells, n = 3 independent experiments), but not completely, by expression of dnIRAK1. Representative experiments are illustrated in Figure 7E and 7F, respectively. In AALEB cells, the dnIRAK1 construct caused an average 86 ± 1% decrease in IL-1β-stimulated NF-κB-driven luciferase activity but did not affect TNFα stimulated activity. These results confirm an important role for IRAK1 in the response to Ps. a. products, presumably via its integral function in the MyD88-dependent portion of the TLR pathway, and strongly suggest that loss or inhibition of IRAK1 is an important component of tolerance.

## Discussion

The host must eradicate pathogens while preventing tissue injury due to inflammation. Repeated exposure of airway epithelium to microbial products is a hallmark of chronic infectious lung diseases but the adaptation has not been previously studied in these key cells. Polarized, well-differentiated hTBE cell cultures used in the current studies recapitulate the morphology and mucus transport function found *in vivo *[39]. We challenged the apical surface of these cultures with soluble products of Ps. a., an important airway pathogen. Prior studies focused on the direct interaction of live Ps. a. bacteria with epithelial cells (see [48] for review). However, bacteria in chronically infected CF lungs are present mostly as intra-luminal masses distal from the airway epithelial surface [49]. Thus, we chose to model the interaction of the apical membrane of polarized hTBE cells with diffusible Ps. a. products, rather than live bacteria. We used late stationary phase TSB cultures of a common strain of Ps. a. instead of clinical isolates. Ps. a. in CF patients frequently become mucoid, produce modified forms of LPS [50] and also secrete quorum sensing molecules [51]. Future studies will be necessary to determine whether modifications of Ps. a. typically found in chronic human infections alter epithelial responses. Although we used non-inoculated TSB processed in parallel as a negative control, we cannot completely exclude that a portion of the stimulation was due to TSB breakdown products induced by bacterial metabolism of the broth. Well-differentiated hTBE cells likely have greater physiologic relevance than cell lines grown directly on plastic. However, well-characterized cell lines facilitate mechanistic studies, so we also used a newly developed airway epithelial cell line (AALEB).

**Figure 8:**
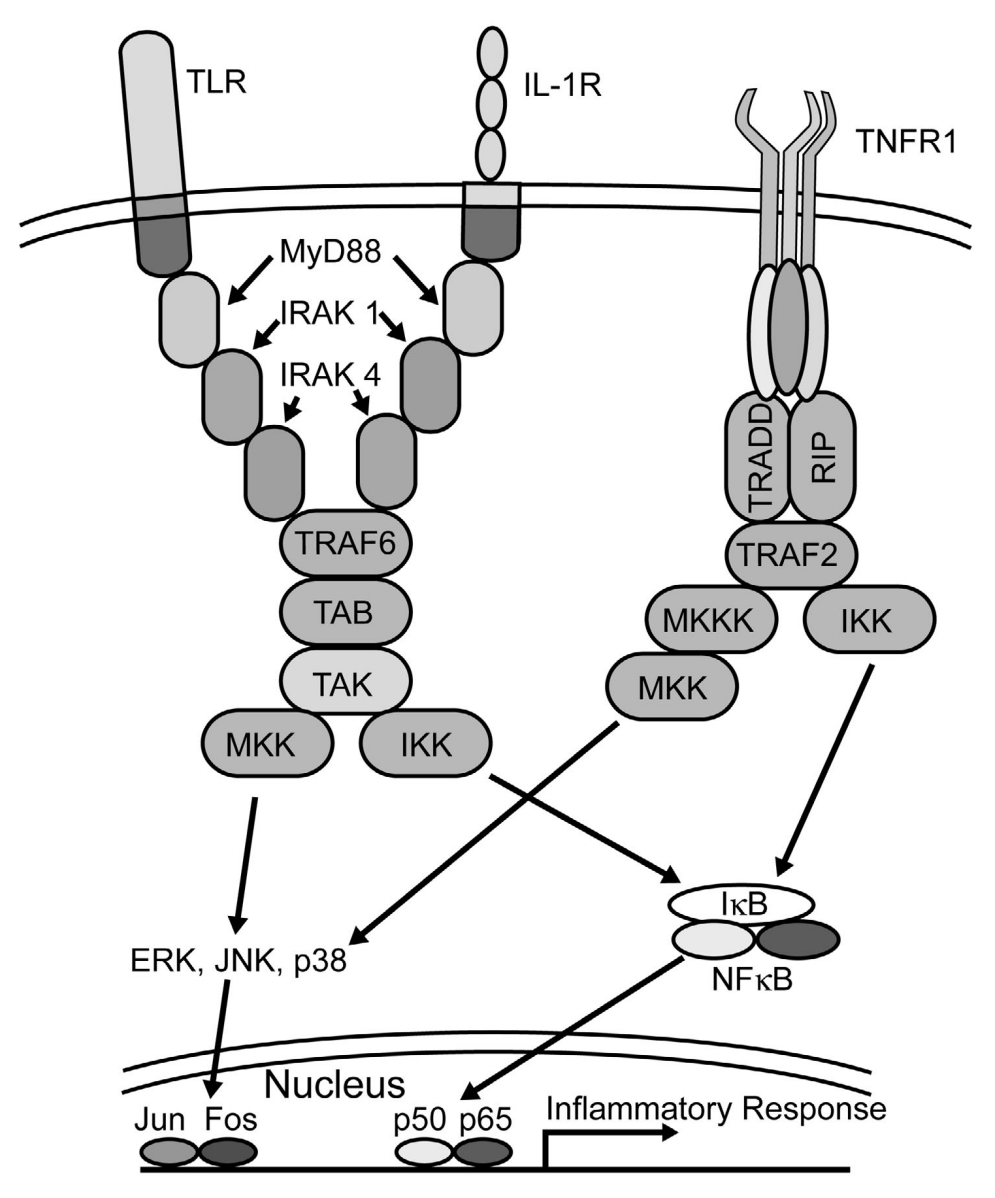
**IRAK1 functions in the TLR and IL-1β pathway upstream of convergence with the TNFα pathway. **A simplified diagram illustrating portions of the TLR, IL-1β and TNFα signalling pathways. Consistent with our observations, disruption of IRAK1 inhibits TLR and IL-1β, but not TNF, activation of NF-κB.

Initial exposure of hTBE or AALEB cells to Ps. a. filtrate induced IL-8 release. This response was preceded by phosphorylation of IRAK1, JNK, p38 and ERK, degradation of IκBα, and generation of NF-κB and AP-1 and transcriptional activity in hTBE cells. IRAK1 phosphorylation leading to activation of NF-κB and AP-1 transcription factors is consistent with activation of the MyD88-dependent portion of the TLR signal transduction pathway. A main finding of the present study in both hTBE and AALEB cells was that cells previously exposed to Ps. a. products for 24 hours had a greatly diminished IL-8 response. Likewise, IRAK1 phosphorylation, IκBα degradation, MAP kinase activation and generation of NF-κB and AP-1 and transcriptional activity after a second Ps. a. exposure were all markedly reduced in Ps. a.-tolerant cells. NF-κB reporter assays strongly suggested that Ps. a.-induced cytokine secretion was transcriptionally regulated and that tolerance was associated with decreased activation of NF-κB transcription factors. The cells were hetro-tolerant to IL-1β, but not TNFα, suggesting that tolerance occurs at a point in common to the TLR and IL-1β pathways, but upstream of the convergence with the TNFα pathway, which is precisely the location of IRAK1 (Figure 8). Ps. a. tolerance was associated with decreased IRAK1 protein content and failure to phosphorylate IRAK1 which is reminiscent of LPS tolerance in other cell types [26-31]. A very recent study demonstrates that macrophage tolerance to the prototypical TLR2 agonist Pam_3_Cys is due to ablation of IRAK1 [52]. Additional evidence for a key role of IRAK1 in the airway epithelial response to Ps. a. was suggested by expression of dnIRAK1, which diminished NF-κB-driven transcription approximately 50%. In AALEB cells, dnIRAK1 did not alter the response to TNFα, but reduced IL-1β effects nearly 90%. Thus, it is likely that factors present in Ps. a. filtrates activate both IRAK1-dependent and -independent pathways leading to NF-κB activation. It is interesting to consider whether tolerant airway epithelial cells were generally "run down" and incapable of responding, but IL-8 release and NF-κB-driven luciferase activity after TNFα treatment of Ps. a.-tolerant cells indicates that the downstream signalling apparatus was fully capable of responding. Components upstream of the convergence of the TLR and TNFα pathways may indeed be "run down", in fact, it is our hypothesis that decreased IRAK1 protein results in Ps. a. tolerance in airway epithelial cells.

Generic IRAK was first recognized as a crucial component of the IL-1β signalling pathway and molecular cloning revealed homology to the Drosophila protein kinase Pelle [4]. More recent studies have revealed 4 IRAK isoforms [53], a critical role for tandem action of IRAK1 and 4 [54] and an inhibitory role for IRAK-M [34]. Ligation of TIR domain-containing receptors induces IRAK1 phosphorylation both auto-catalytically and by unidentified kinases. Phosphorylated IRAK1 becomes degraded by proteasomes in cells stimulated by IL-1β but not TNFα, which correspondingly results in desensitization of the IL-1β, but not TNFα response [55]. The precise mechanisms regulating IRAK1 localization and function during the propagation and termination of the TLR signal in hTBE cells are not fully understood, and require further study.

While the kinetics of the initial response of naive cells suggested a direct interaction of Ps. a. products to trigger the TLR pathway, airway epithelial cells at 24 hours were likely exposed to released autocrine/paracrine factors that may have also contributed to tolerance. However, this was probably not mediated by IL-1β, since this cytokine was consistently undetectable in hTBE cell media after Ps. a. stimulation (SHR and MWV unpublished observations). Moreover, such factors would have to selectively target the upstream TLR/IL-1β, pathway since TNFα responses were not affected.

Ps. a. is a complex and adaptable bacterium that secretes many factors known to damage host cells and/or induce inflammation such as pilin, flagellin, pyocyanin, hemolysins, autoinducer, LPS, proteases, and small unidentified heat-stable factors [9,40,50,56-59]. There is a broad array of mechanisms by which these agents may act, including TLR activation, generalized oxidative stress due to redox cycling of pyocyanin, and proteolytic modification of target cells by Ps. a. elastase or alkaline protease. IRAK activation in a human bronchial cell line has been shown following neutrophil elastase [60]. Rapid activation of IRAK1, in conjunction with desensitization of the IL-1β but not the TNFα response, suggests that Ps. a. products directly trigger an MyD88-dependent TLR pathway. Activation of airway epithelial cells by a variety of live bacteria and bacterial products is TLR2-dependent [10,12-14]. Polymyxin B treatment is commonly used to block LPS effects [61], but it did not reduce Ps. a.-stimulated hTBE cell IL-8 secretion, and Ps. a. derived LPS is a poor inducer of IL-8 in these cells (SHR and MWV unpublished observations), suggesting that LPS stimulation of TLR4 was not predominant. Further studies are needed to chemically identify the most quantitatively important TLR pathway-stimulating substance(s) produced by *Pseudomonas aeruginosa*. In this regard, recent studies indicate a potential role for TLR2 activation by lipopeptides encoded by Ps. a. genes [62].

While controversial, several lines of evidence suggest that inflammatory responses in the CF airway may be intrinsically enhanced or protracted due to the absence of functioning CFTR (see [11] and references therein). Thus, examination of differences in the development of tolerance in CF cells is worthy of further scrutiny. Although hTBE cells in our *in vitro *model became tolerant to Ps. a., chronically infected airways in CF humans are typically severely inflamed. Whether tolerance exists *in vivo*, if it is helpful or harmful, different in CF, or if it is overwhelmed by the heavy bacterial burden are important, but unanswered questions. Multiple cell types, which are constantly turning over, contribute to *in vivo *responses. Bacterial products may penetrate beyond the epithelial barrier to trigger inflammation or systemic responses. We speculate that epithelial tolerance to Ps. a. occurs *in vivo*, and that inflammation would be even more severe in its absence. However, further clinical and/or animal model studies are needed to address these key questions.

## Conclusion

We have shown that exposure of primary, well-differentiated hTBE cells to Ps. a. products causes selective tolerance. Ps. a. products elicit a response via the TLR signal transduction pathway that becomes down-regulated, likely due to decreased IRAK1 protein content and inhibition of IRAK1 phosphorylation. A greater understanding of the precise mechanisms decreasing airway epithelial cell generation of inflammatory mediators will suggest new avenues for anti-inflammatory therapies to minimize lung destruction typical of CF and other chronic infectious airway diseases.

## Authors' contributions

QW conceived the studies, performed culture challenges, most of the biochemical assays, and drafted the manuscript. ZL cloned the DD domain of IRAK-1 into the viral vector, characterized the response of AALEB cells to Ps. a. and performed all aspects of the reporter assays on both AALEB and hTBE cells. MWV performed specific biochemical studies, most of the statistical analysis and critically contributed to the experimental design and written manuscript. SHR participated in the design, coordination and analysis of the experiments, performed western blots, prepared the figures, and wrote and submitted the final manuscript. All authors read and approved the final manuscript.
